# Efficacy of a novel proprietary dietary supplement (TRI 360^TM^) on psychological symptoms and stress-related quality of life in adult subjects: A randomized controlled clinical trial

**DOI:** 10.3389/fpsyt.2022.919284

**Published:** 2022-08-11

**Authors:** Mahendra Kumar Trivedi, Alice Branton, Dahryn Trivedi, Sambhu Mondal, Snehasis Jana

**Affiliations:** ^1^Trivedi Global, Inc., Henderson, NV, United States; ^2^Trivedi Science Research Laboratory Pvt. Ltd., Thane, India

**Keywords:** TRI 360^TM^, dietary intervention, mental stress, Psychological Questionnaire Scoring, klotho, tumor necrosis factor-alpha, vitamin D_3_ metabolite, 17-β-estradiol

## Abstract

Nowadays, diet plays an increasingly important role in normal physiology and mental health. Recently, many studies have shown that more use of dietary supplements in mental and psychological disorders. Study objective was to investigate safety and efficacy of proprietary nutraceutical combination (TRI 360^TM^) on psychological symptoms in adult human subjects with one or more psychological symptoms in open-label, single-center, parallel-group, randomized controlled trial. Eighty-four participants aged 20–45 years with psychological symptoms were completed this trial. Participants were randomly assigned to placebo and treatment groups. Treatment group received TRI 360^TM^ capsules twice a day. TRI 360^TM^ was well-tolerated and didn't show treatment-related adverse-events upto 180 days. All assessed perception scorings on psychological symptoms like fatigue, mental stress, sleep disturbance, anxiety, depression, emotional trauma, mood changes, self-confidence, willpower, and motivation were very significantly (*p* ≤ 0.0001) improved in TRI 360^TM^ participants than placebo control group. Furthermore, significantly (*p* ≤ 0.001) increased levels of functional biomarkers: vitamin C and D_3_ metabolites, neurotransmitters, hormones, antiaging protein (klotho) level; and decreased proinflammatory cytokines and oxidative stress marker, malondialdehyde in TRI 360^TM^ group than placebo. According to these findings, the use of TRI 360^TM^ supplementation as a potentially safe therapeutic option for reducing psychological symptoms in healthy adults.

## Introduction

Mental health disorders are progressively recognized as a major public health burden. World Health Organization (WHO) study reported that mental disorders are categorically estimated to be five out of the ten leading causes of disability. WHO also reported that the promotion of mental health and the prevention of mental disorders can help maintain a positive impact on quality of life, which can be economically beneficial (1). In general practice, mental health studies have found that the most common diagnoses were anxiety, depression, neurasthenia, mood changes, and fatigue problems with alcohol (2). The most prevalent mental disorders in numerous countries are depression, bipolar disorder, schizophrenia, obsessive-compulsive disorder (OCD), attention deficit hyperactivity disorders (ADHD), lack of decision-making ability, low willpower, lack of motivation, inspiration, enthusiasm, ambition, etc. The intake of dietary supplements in Asian and American countries reflects that they are often deficient in nutrients like vitamins, minerals, omega-3 fatty acids, etc. (3). Mental disorders are commonly associated with nutritional deficiencies of omega-3 fatty acids, B vitamins, minerals, and amino acids that are precursors to neurotransmitters (4). The last 90 years of research demonstrate the importance of dietary nutrients (B vitamins) for mental health like irritability and mood problems (5). Apart from vitamins and minerals, ginseng extract have a crucial impact on various biological processes like epigenetic modification of gene expression and plasticity of neurons, synapses, and neural networks that ultimately reflects on the change of moods and behavior. Hormonal abnormalities may arise at endogenous mediators' synthesis, metabolism or receptor levels (6).

Stress has been linked to poor mental health (7), and is associated with poorer neurocognitive performance in all-aged peoples (8). Stress can induce poor productivity, irritability, mind chattering, poor cognition, low level of enthusiasm, and chronic fatigue (9). Due to these factors, there is a reduction in the overall quality of life and economic loss (10). In depressed patients, altered redox status is noted in the central nervous system (CNS) and oxidative disturbance is associated with lowered concentrations of several endogenous antioxidant enzymes (11). Pharmacological treatments are partially proven to be effective in treating from moderate to severe symptoms in major depressive disorders (MDD), have only modest effect sizes and approximately 40% of psychiatric patients do not respond adequate relief to the existing drug therapies. Moreover, antipsychotics are associated with severe side effects and a propensity for abuse (12). Nutritional psychiatry examines the role of diet and nutrition in mental health from a variety of perspectives (13). There are several mechanisms by which diet and its components may affect chronic stress, anxiety, depression, mood disorders, fatigue, etc. They include modulating inflammatory pathways, the oxidative stress pathway, epigenetic pathways, mitochondrial dysfunction, the gut microbiota, signal transduction pathways, and neurogenesis pathways (14). A few randomized clinical trials (RCTs) confirmed the benefits of a Mediterranean-style diet on mental health in depression (15). Based on the existed literature, low levels of vitamin C and D_3_ active metabolites (25-OH vitamin D_3_ and 1, 25-(OH)_2_ vitamin D_3_) (16, 17), hormones (oxytocin, 17-β-estradiol, and insulin) (18–20), neurotransmitters (noradrenaline, acetylcholine, and dopamine) (21–23), and klotho protein (24) would be associated with psychological, emotional, and mental health symptoms (*i.e.*, anxiety, depression, sleep disorders, stress, mental restlessness, and emotional trauma). Higher levels of proinflammatory cytokines (TNF-α, IL-1β, and IL-8) (25) and oxidative stress markers (MDA and oxidized-LDL) would be associated with psychological and mental health symptoms (26, 27).

TRI 360^TM^ is a novel proprietary dietary supplement formulation of vitamin A, as beta carotene, vitamin B_6_, vitamin B_12_, vitamin C, vitamin D_3_, vitamin E, calcium chloride, iron sulfate, magnesium gluconate, zinc chloride, sodium selenate, and ginseng powder. These compounds have reported some clinical efficacy in individuals with mental disorders when used either alone and/ or mixed formulation, and did not show the side effects usually associated with conventional antidepressants (28). Theoretically, consuming complex, multi-nutrient formulations of herbal extracts, vitamins, and minerals should be safe, since most preparations contain nutrients that have been part of the human diet for millennia, and at safe levels that are determined by the Dietary Reference Intakes (DRI). However, the safety profile of commercial TRI 360^TM^ formulae may differ from foods because of the amounts and combinations of nutrients they contain. There is a need for direct evaluation of safety and tolerability for this complex formula (TRI 360^TM^) as this is being studied and used clinically with increasing frequency. The aim of the current study was to evaluate the safety and efficacy of TRI 360^TM^ for the relief of psychological symptoms in adult subjects in a randomized controlled clinical trial. This clinical trial sought to evaluate whether TRI 360^TM^ supplementation can modulate various functional physiological biomarkers in human. We hypothesized that nutraceutical combination capsules supplementation treatment would lead to the improvement of perceived psychological scoring/symptoms that can correlate with changes in levels of vital functional physiological biomarkers in serum such as vitamins (25-OH vitamin D_3_, 1, 25-dihydroxy vitamin D_3_, and vitamin C), oxidative stress biomarker (oxidized-LDL and malondialdehyde), hormones (oxytocin, 17-β-estradiol, and insulin), neurotransmitters (acetylcholine, noradrenaline, and dopamine), pro-inflammatory cytokines (TNF-α, IL-1β, and IL-8), and antiaging biomarker (klotho protein).

## Methods

### Study design/sample size

**Figure 1:**
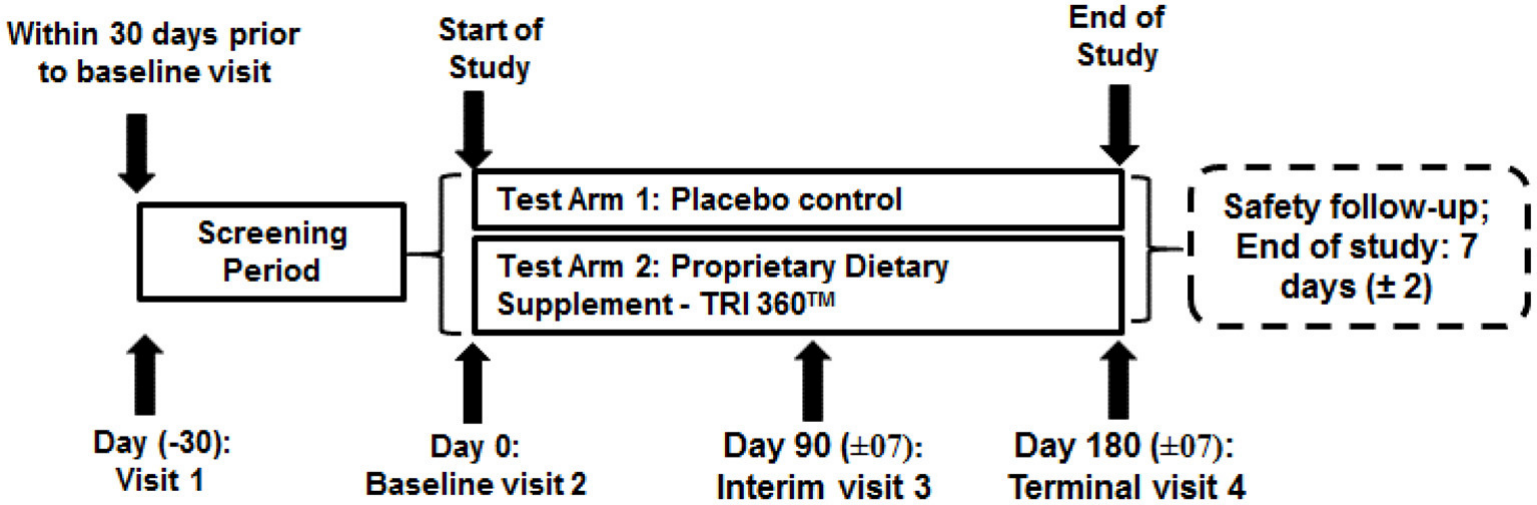
Schematic diagram of study design.

This study involved a randomized, active-controlled, open-label, single-center, parallel-group design to investigate the safety and efficacy of the proprietary dietary supplement (TRI 360^TM^) with placebo in improving physiological function and health wellness in adult subjects. After evaluation of screening parameters and receipt of a signed informed consent, eligible subjects were randomly assigned to the treatment and placebo control group (1:1) with the help of simple randomization technique (allocation concealment mechanism) using SAS software (Version: 9.4 or higher; SAS^®^ Institute Inc., Cary, NC, USA) to generate the random allocation sequence. An a priori power analysis was undertaken to estimate the required sample size. We predicted a Cohen's d effect size of 0.8 for the treatment group. Assuming a power of 80%, a type 1 error rate (alpha) of 5%, and a 10% drop-out rate, the total number of participants to find an effect was estimated as 104. Enrolled participants were assigned into two groups (proprietary TRI 360^TM^ or placebo control) using SAS software. Total 104 subjects were screened, out of which 84 subjects were enrolled and divided into two groups. Subjects reported to the study site for the following visits—visit 1: screening visit (up to 30 days prior to randomization), visit 2: randomization/baseline/ visit (day 0), visit 3: interim visit day 90 (±7 days), and visit 4: terminal visit day 180 (±7 days). Safety follow-up for 1 week (±2 days) after the end of treatment (Figure 1).

### Inclusion criteria

Individuals who meet all the following criteria were included as appropriate participants in the trial: 1. Male or female aged between 20 to 45 years at the time of consent. 2. Subjects with skin reflection > 6% measured *via* advanced glycation end products (AGEs) reader during screening. 3. Subjects with one or more complaints of the following symptoms: asthenia (general weakness/tiredness/fatigue), sleep disturbances (poor quality sleep), anxiety/depression/post-traumatic stress disorder (PTSD), stress and confusion, mental restlessness/mind chattering, fear from the future/ongoing negative thoughts, emotional trauma, lack of self-worth, hopelessness / suicidal tendencies attention deficit disorders (ADD)/ attention deficit hyperactivity disorders (ADHD), low libido, menstrual disorder (female subjects): cramps, heavy bleeding, bleeding for a long duration, bloating, floating, back pain, mood swings, premenstrual syndrome, low confidence/low willpower/inability, lack of inspiration/motivation/enthusiasm. 4. Body mass index (BMI): 18.5 to 30.0 kg/m^2^, both inclusive. BMI calculated as weight in kg / (height in meters) 5. Females of childbearing age agreed to use an acceptable birth control during the study. 6. Agreed to provide written informed consent and followed the study directions to participate in the study and complete all follow-up visits. 7. Able to comply with the study requirements and procedures as per protocol. 8. All subjects were judged for eligibility by the principal investigator, or sub-investigator, or physician during a pre-study screening assessment.

### Exclusion criteria

Participants were ineligible if they had met any of the following conditions: History of allergic responses/hypersensitivity to any of the ingredients of the study product. History within last 1 year or currently having alcohol dependence or drug abuse. Significant diseases or clinically significant abnormal findings in medical history, physical examination, laboratory evaluations etc., during screening. Regular vigorous aerobic/endurance exercise (>3 vigorous bouts/week). Subjects taking any treatment for the complaints mentioned in the inclusion criteria no. 3. Known history of positive human immune virus (HIV), hepatitis C virus (HCV), hepatitis B surface antigen (HBsAg), or venereal disease research laboratory test (VDRL)/ rapid plasma reagin (RPR). Subjects with non-healthy, non-homogenous, damaged over the skin at the measuring are used during AGE reader. Subjects with birthmarks or excessive hair over the skin at the measuring area used during AGE reader can influence the results. Subjects with the usage of self-tanning agents for at least 10 days before screening. Female subjects who demonstrate a positive pregnancy test or are currently breastfeeding or planning pregnancy. Any other acute or chronic illness could compromise the integrity of study data or place the subject at risk by participating in the study.

### Composition, rational, and dose regimen of TRI 360^TM^

Each capsule of TRI 360^TM^ consisted of vitamin A, as beta carotene (833 IU), vitamin B_6_ (0.75 mg), vitamin B_12_ (3.5 μg), vitamin C (25 mg), vitamin D_3_ (400 IU), vitamin E (11 IU), calcium chloride (50 mg), iron sulfate (3.5 mg), magnesium gluconate (20 mg), zinc chloride (5 mg), sodium selenate (12.5 μg), and ginseng powder (100 mg). The available literature data, preclinical data, and recommended daily allowance (RDA) intake in humans of each component of TRI 360^TM^ were all considered to select the doses for the study. Each ingredient of TRI 360^TM^ is safe for consumers/humans and under GRAS (Generally Regarded as Safe)^1^. The dose of each ingredient was considered as per the NIH-RDA guidelines. Subjects were instructed to take the TRI 360^TM^ capsule twice a day, one in the morning and one in the evening, preferably with meals.

### Withdrawal criteria

Participants were requested for withdrawal from the study if they fulfilled the following criteria: poor compliance (mean compliance<85% at the last estimation) or non-compliance, the occurrence of a severe adverse effect. No subject was discontinued in the placebo and TRI 360^TM^ groups.

### Brief methodology

Screening was done within 30 days before randomization. After obtaining the informed consent, subjects were allowed to participate in the study-related screening activities. The subjects randomized to the placebo group did not give any product. The subjects were evaluated for general health, adverse effects, psychological questionnaires, and other serum physiological biomarkers at each visit. The primary outcome measure was safety. At each visit, the efficacy was assessed by patient-reported psychological questionnaires, symptoms related to adverse effects, and biomarkers levels in blood/serum. Secondary outcomes were analyzed with respect to different biomarkers related to health and wellness parameters. These includes vitamins (25-OH vitamin D_3_, 1, 25-dihydroxy vitamin D_3_, and vitamin C), oxidative stress biomarker (oxidized-LDL and malondialdehyde), hormones (oxytocin, 17-β-estradiol, and insulin), neurotransmitters (acetylcholine, noradrenaline, and dopamine), pro-inflammatory cytokines (TNF-α, IL-1β, and IL-8), and antiaging biomarker (klotho protein) were analyzed with the help of standard in-house protocol as per manufacturer's instructions.

### Adverse effects

Safety was determined by monitoring adverse effects (AEs), which were classified using the latest version of MedDRA (Medical Dictionary for Regulatory Activities) terminology^2^. A complete medical history, including a comprehensive review of all current and past diseases and their respective treatments, was taken prior to starting study therapy. The investigator or sub-investigator performed a physical examination including vital signs (blood pressure, pulse rate, respiratory rate, and temperature) at the time of screening, day 0, 90, and 180 visit, to evaluate the adverse effects if any. Subjects were instructed to remain seated for about 5 minutes before vital signs were obtained. Females of childbearing potential had a negative pregnancy test performed during these visits (day 0, 90, and 180). Specific interviews monitored AEs during day 0, 90, and 180 visits by physical presence and/or phone calls between visits by contacting a clinical research coordinator or the study investigators.

### Perceived changes of psychological symptoms

Perceived changes for symptoms/complaints were assessed based on Psychological Questionnaire Scoring (PQS). Questionnaire-based evaluation of all the symptoms mentioned in inclusion criteria no. 3 were evaluated at day 0, 90, and 180, according to the relevant scale of scoring with slight modifications (29–32).

### Blood sampling and serum preparation

Blood samples were collected at day 0, 90, and 180 visits. Serum was prepared using standard method by LabCorp (Salt Lake City, Utah, USA). The collected serum samples were frozen at −20°C until all biomarkers analysis. All biomarkers levels were determined by parameter-specific standard ELISA methods as per manufacture's instructions.

### Assessment of physiological biomarkers

Physiological biomarkers were assessed in serum using in-house protocol as per manufacturer's instructions. 17-β-estradiol (CAT#B7K720) and 25-OH vitamin D_3_ (CAT#B5P020) ELISA-based kits were obtained from Abbott Diagnostics, USA and measured using Architect ci 4100, Abbott Diagnostics, USA. TNF-α (CAT#558273), IL-1β (CAT#558279), IL-8 (CAT#558277) ELISA-based kits were obtained from BD Biosciences, USA and measured by FACS Calibur, BD Biosciences, USA. Vitamin C (CAT#E-EL-0011), 1, 25 dihydroxy vitamin D (CAT#E-EL-0016), oxytocin (CAT#E-EL-0029), klotho (CAT#E-EL-H5451), oxidized LDL (CAT#E-EL-H0124), malondialdehyde (CAT#E-EL-0060), norepinephrine (CAT#E-EL-0047), dopamine (CAT#E-EL-0046), and acetylcholine (CAT#E-EL-0081) ELISA-based kits were obtained from Elabscience Biotechnology Inc. USA and determined by SpectraMax 190/SpectraMax M2e, Molecular Devices, USA.

### Statistical analysis

The psychological data were represented as mean ± Standard deviation (SD) and subjected to statistical analysis using SAS^®^ 9.4 (SAS Institute Inc., Cary, USA). Psychological scoring parameters were analyzed within the treatment using paired *t*-test and the between the treatments analysis was performed using Analysis of Covariance (ANCOVA) with treatment as fixed effect and baseline as covariate to estimate 95% confidence interval of the difference between treatments. For physiological biomarker analysis data were represented as mean ± standard error of the mean (SEM), between two group's pair Student's *t*-test was used for statistical assessment (Sigma-Plot V11.0). The *p* ≤ 0.05 was considered as statistically significant.

## Results

### Subject disposition and adverse effects assessment

**Figure 2:**
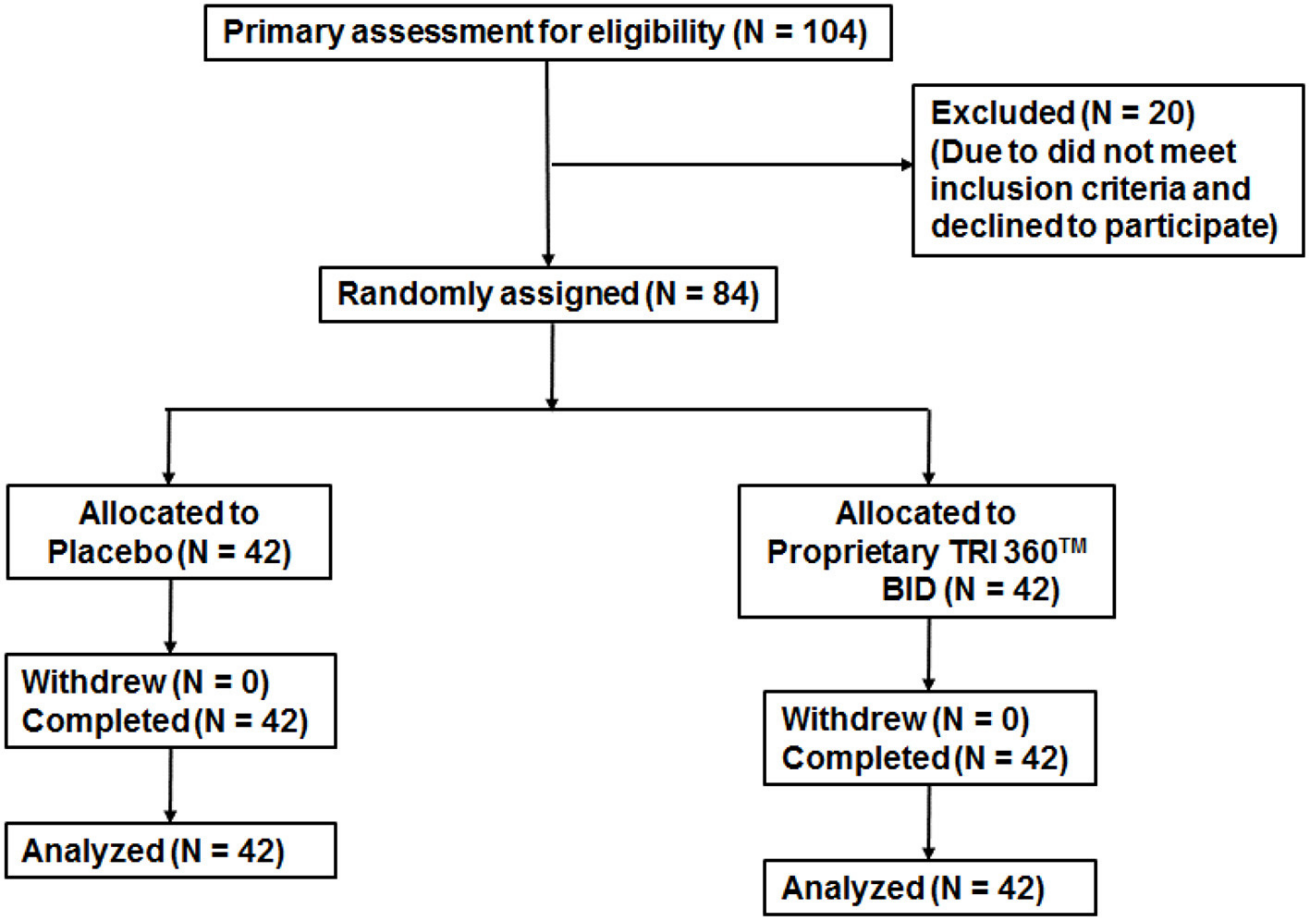
Trial flow chart of subject disposition Primary recruitment identified 104 volunteers. After excluding 20 ineligible persons the authors randomly assigned 84 participants as follows: placebo (*n* = 42) and proprietary TRI 360^TM^ (*n* = 42) one capsule in the morning and one capsule in the evening.

A total of 104 subjects were screened, out of which 84 subjects were enrolled in the study. Among which, 42 (24 male + 18 female) subjects were assigned to the placebo control group, and 42 (24 male + 18 female) subjects were allocated to the proprietary TRI 360^TM^ group (Figure 2). No adverse events (AEs) were reported during the study period. Demographic characteristics of study subjects were recorded. There were no clinically significant abnormalities in physical findings like body weight, body mass index values observed between the two groups from baseline to follow-up visit (Table 1).

**Table 1:** Summary of the baseline demographic and clinical baseline characteristics.

**Demographic and baseline characteristics**	**Placebo control** **(*N =* 42)**	**TRI 360^TM^** **(*N =* 42)**
**Age (Years)**		
Mean ± SD	34.7 ± 6.42	33.4 ± 5.86
Median	37	35
Min, max	20, 44	20, 44
**Sex [*****n*** **(%)]**		
Male	24 (57.14)	24 (55.27)
Female	18 (42.86)	18 (41.53)
**Race [*****n*** **(%)]**		
Asian	42 (100)	42 (100)
White	0 (0.00)	0 (0.00)
Black or African American	0 (0.00)	0 (0.00)
American Indian or Alaska native	0 (0.00)	0 (0.00)
Native Hawaiian or other pacific Islander	0 (0.00)	0 (0.00)
Other	0 (0.00)	0 (0.00)
**Height (cm)**		
Mean ± SD	161.0 ± 8.50	159.7 ± 7.84
Median	162.5	160
Min, max	142, 178	141, 175
**Weight (kg)**		
Mean ± SD	63.39 ± 10.926	62.84 ± 11.245
Median	61.65	61.6
Min, Max	46.8, 86.0	43.0, 89.3
**BMI (kg/m** ^**2**^ **)**		
Mean ± SD	24.40 ± 3.565	24.27 ± 3.482
Median	24.25	24.34
Min, max	18.6, 29.8	18.5, 28.9
**Marital status [*****n*** **(%)]**		
Married	37 (88.10)	36 (86.28)
Unmarried	5 (11.90)	6 (13.27)
**Literacy status [*****n*** **(%)]**		
Literate	40 (95.24)	39 (94.56)
Illiterate	2 (4.76)	3 (4.37)
**Substance usage [*****n*** **(%)]**		
**Alcohol**		
Previous	4 (9.52)	4 (9.18)
Current	4 (9.52)	0 (0.00)
Never	34 (80.95)	38 (84.25)
**Cigarettes/biddies**		
Previous	0 (0.00)	1 (2.60)
Current	1 (2.38)	1 (2.60)
Never	41 (97.62)	40 (94.59)
**Tobacco**		
Previous	3 (7.14)	4 (2.70)
Current	10 (23.81)	8 (13.51)
Never	29 (69.05)	30 (83.78)

BMI, Body Mass Index; SD, Standard Deviation; N, number of subjects in the specified treatment arm; n, number of subjects in the specified category; Percentages were based on the number of subjects in the specified treatment arm.

### Psychological symptoms

Psychological symptoms/scores did not show any significant difference between the treatment and placebo control groups during baseline measurement (prior to experiment, at day 0). However, at visit 3 (day 90), there was a significant reduction of the perceived psychological symptoms/scores such as asthenia, −1.881 ± 0.8677, *p* < 0.0001, 95% confidence interval (CI) (−2.26, −1.50); sleep disturbances, −1.905 ± 1.0766, *p* < 0.0001, 95% CI (−2.37, −1.44); anxiety/depression/PTSD, −1.690 ± 0.8377, *p* < 0.0001, 95% CI (−2.05, −1.33); stress and confusion, −1.500 ± 0.9592; *p* <0.0001, 95% CI (−1.92, −1.08); mental restlessness, −1.548 ± 0.9526, *p* <0.0001, 95% CI (−1.96, −1.13); future fearness, −1.571 ± 1.1375, *p* <0.0001, 95% CI (−2.07, −1.08); emotional trauma, −1.738 ± 1.1222, *p* <0.0001, 95% CI (−2.23, −1.25); lack of self–worth score, −1.690 ± 0.9683, *p* <0.0001, 95% CI (−2.11, −1.27); hopelessness/suicidal ideation, −1.690 ± 0.9683, *p* <0.0001, 95% CI (−2.11, −1.27); ADD/ADHD, −1.381 ± 0.9759, *p* <0.0001, 95% CI (−1.80, −0.96); libido/sexual disireness, −1.548 ± 1.4258, *p* <0.0001, 95% CI (−2.17, −0.93); menstrual cycle disorders/mood disorders symptoms, −1.667 ± 1.5392, *p* <0.0001, 95% CI (−2.71, −0.62);]; lack of confidence/willpower, −1.333 ± 1.0465, *p* <0.0001, 95% CI (−1.79, −0.88); and lack of inspiration/motivation/enthusiasm/ambition, −1.952 ± 1.1486, *p* <0.0001, 95% CI (−2.45, −1.45) for the TRI 360^TM^ group as compared with the placebo control group.

Similarly, at visit 4 (day 180), there was a significant reduction the score of asthenia, −2.476 ± 0.9383, *p* < 0.0001, 95% CI (−2.88, −2.07); sleep disturbances, −2.738 ± 1.0981, *p* < 0.0001, 95% CI (−3.21, −2.26); anxiety/depression/PTSD, −2.214 ± 0.9102, *p* < 0.0001, 95% CI (−2.61, −1.82); stress and confusion, −1.738 ± 0.9932; *p* <0.0001, 95% CI (−2.17, −1.31); mental restlessness, −1.976 ± 1.0632, *p* <0.0001, 95% CI (−2.44, −1.51); future fearness, −2.143 ± 0.9848, *p* <0.0001, 95% CI (−2.57, −1.72); emotional trauma, −2.048 ± 1.0712, *p* <0.0001, (−2.51, −1.58); lack of self–worth score, −2.643 ± 1.0259; *p* <0.0001, 95% CI (−3.09, −2.20); hopelessness/suicidal ideation, −2.524 ± 1.1179, *p* <0.0001, 95% CI (−3.01, −2.04); ADD/ADHD, −1.976 ± 1.1065, *p* <0.0001, 95% CI (−2.46, −1.49); libido/sexual disireness, −2.310 ± 1.1628, *p* <0.0001, 95% CI (−2.81, −1.80); menstrual/mood disorders symptoms, −2.944 ± 1.5619, *p* <0.0001, 95% CI (−4.00, −1.89);]; lack of confidence, −2.095 ± 1.0990, *p* <0.0001, 95% CI (−2.57, −1.62); and lack of inspiration/motivation/enthusiasm, −1.952 ± 1.1486, *p* <0.0001, 95% CI (−2.45, −1.45) for the TRI 360^TM^ group compared with the placebo control group (Table 2).

**Table 2:** Assessment of psychological symptoms after treatment with the proprietary dietary supplement (TRI 360^TM^) capsules in human subjects, at day 90 and 180 visits.

**Parameter**	**Visit (Day)**	**Compari-son**	**Mean ±SD**	**95 % CI**	***p*-value**
Asthenia	90	Placebo Control	−1.881 ± 0.8677	(−2.26, −1.50)	<0.0001
	180	*vs*.	−2.476 ± 0.9383	(−2.88, −2.07)	<0.0001
Sleep disturbances	90	TRI 360^TM^	−1.905 ± 1.0766	(−2.37, −1.44)	<0.0001
	180		−2.738 ± 1.0981	(−3.21, −2.26)	<0.0001
Anxiety/depression /PTSD	90		−1.690 ± 0.8377	(−2.05, −1.33)	<0.0001
	180		−2.214 ± 0.9102	(−2.61, −1.82)	<0.0001
Stress and confusion	90		−1.500 ± 0.9592	(−1.92, −1.08)	<0.0001
	180		−1.738 ± 0.9932	(−2.17, −1.31)	<0.0001
Mental restlessness	90		−1.548 ± 0.9526	(−1.96, −1.13)	<0.0001
	180		−1.976 ± 1.0632	(−2.44, −1.51)	<0.0001
Future fear	90		−1.571 ± 1.1375	(−2.07, −1.08)	<0.0001
	180		−2.143 ± 0.9848	(−2.57, −1.72)	<0.0001
Emotional trauma	90		−1.738 ± 1.1222	(−2.23, −1.25)	<0.0001
	180		−2.048 ± 1.0712	(−2.51, −1.58)	<0.0001
Lack of self-worth	90		−1.690 ± 0.9683	(−2.11, −1.27)	<0.0001
	180		−2.643 ± 1.0259	(−3.09, −2.20)	<0.0001
Hopelessness/suicidal ideation	90		−1.714 ± 1.1096	(−2.20, −1.23)	<0.0001
	180		−2.524 ± 1.1179	(−3.01, −2.04)	<0.0001
ADD/ADHD (Inability to focus)	90		−1.381 ± 0.9759	(−1.80, −0.96)	<0.0001
	180		−1.976 ± 1.1065	(−2.46, −1.49)	<0.0001
Libido/sexual disireness	90		−1.548 ± 1.4258	(−2.17, −0.93)	<0.0001
	180		−2.310 ± 1.1628	(−2.81, −1.80)	<0.0001
Menstrual/mood disorders symptoms	90		−1.667 ± 1.5392	(−2.71, −0.62)	<0.0001
	180		−2.944 ± 1.5619	(−4.00, −1.89)	<0.0001
Lack of confidence/willpower/inability	90		−1.333 ± 1.0465	(−1.79, −0.88)	<0.0001
	180		−2.095 ± 1.0990	(−2.57, −1.62)	<0.0001
Lack of inspiration/motivation/enthusiasm	90		−1.500 ± 1.2180	(−2.03, −0.97)	<0.0001
	180		−1.952 ± 1.1486	(−2.45, −1.45)	<0.0001

Data are represented as mean ± SD. Placebo group (n = 42) and TRI 360^TM^ group (n = 42). Data were analyzed and p-value is calculated using two-sample t-test. At the end of study, subjects present in the placebo group (n = 42) and TRI 360^TM^ group (n = 42).

SD, Standard deviation; CI, Confidence interval; PTSD, Post-traumatic stress disorder; ADD, Attention deficit disorders; ADHD, Attention deficit hyperactivity disorders.

### Functional physiological biomarkers in serum

#### Vitamins

The level of vitamin D_3_ active metabolite, 25-OH vitamin D_3_ in the placebo control group was 10.91 ± 0.47 ng/mL, which was increased significantly (F_(2, 123)_ = 28.885, *p* ≤ 0.001) by 61.23% (at day 90) and 71.31% (at day 180) in the TRI 360^TM^ group as compared to the placebo control group. Further, serum concentration of 1, 25-(OH)_2_ vitamin D_3_ in the placebo control group was 124 ± 2.85 ng/mL, which was also increased significantly (F_(2, 123)_ = 105.866, *p* ≤ 0.001) by 137.74% (at day 90) and 156.96% (at day 180) in the TRI 360^TM^ group as compared to the placebo control group. In addition, the level of ascorbic acid (vitamin C) was significantly (F_(2, 123)_ = 18.514, *p* ≤ 0.001) increased by 85.02% (at day 90) and 87.77% (at day 180) in the TRI 360^TM^ group with respect to the placebo group (17.42 ± 0.74 μg/mL).

#### Oxidative stress biomarkers

The oxidative stress biomarker, malondialdehyde (MDA), was measured in serum significantly (F_(2, 123)_ = 58.404, *p* ≤ 0.001) decreased by 61.13% (at day 90) and 52.03% (at day 180) in the TRI 360^TM^ group as compared to the placebo control group. The level of oxidized LDL was decreased significantly (F_(2, 123)_ = 26.545, *p* ≤ 0.001) by 43.70% (at day 90) and 42.42% (at day 180) in the TRI 360^TM^ group as compared to the placebo control (963.55 ± 43.17 pg/mL) group.

#### Hormones

Tukey's *post-hoc* analysis revealed that the level of oxytocin was significantly (F_(2, 123)_ = 78.446, *p* ≤ 0.001) increased by 331.55% (at day 90) and 310.16% (at day 180) in the TRI 360^TM^ group as compared to the placebo control (88.05 ± 6.39 pg/mL) group. Further, pair *t*-test analysis showed that 17-β-estradiol level was non-significantly (CI = −104.213 to 4.673; *t* = −1.819, *p* = 0.073) increased by 51.27% in the TRI 360^TM^ group at day 180 compared to the placebo group. Insulin level was non-significantly (F_(2, 123)_ = 1.325, *p* = 0.270) increased by 83.29% and 51.82% at day 90 and 180 visits, respectively in the TRI 360^TM^ group than placebo.

#### Antiaging protein

Besides, Tukey's *post-hoc* analysis exhibited the expression of an antiaging biomarker, klotho protein was significantly (F_(2, 123)_ = 141.674, *p* ≤ 0.001) increased by 371.56% (at day 90) and 500.89% (at day 180) in the TRI 360^TM^ group with respect to the placebo group (2.25 ± 0.04 pg/mL) (Table 3).

**Table 3:** Measurement of serum biomarkers related to vitamins, oxidative stress, hormones, and aging in serum after treatment with the proprietary dietary supplement (TRI 360^TM^) capsules in human subjects, measured at days 90 and 180.

**Parameter**	**Placebo** **(Mean ±** **SEM)**	**TRI 360** ^TM^	
		**Day 90** **(Mean ±** **SEM)**	**Day 180** **(Mean ±** **SEM)**
**Vitamin**			
25-OH Vitamin D_3_ (ng/mL)	10.91 ± 0.47	17.59 ± 0.89***	18.69 ± 0.91***
1, 25 Dihydroxy Vitamin D_3_ (pg/mL)	124 ± 2.85	294.80 ± 14.17***	318.63 ± 10.51***
Vitamin C (μg/mL)	17.42 ± 0.74	32.23 ± 2.99***	32.71 ± 1.66***
**Oxidative stress biomarker**			
Malondialdehyde (ng/mL)	*3, 346.63*±124.73	*1, 300.69*±57.08***	*1, 605.50*±209.21***
Oxidized-LDL (pg/mL)	963.55 ± 43.17	542.44 ± 47.52***	554.84 ± 48.66***
**Hormone**			
Oxytocin (pg/mL)	88.05 ± 6.39	379.98 ± 24.41***	361.15 ± 19.60***
17-β-estradiol (ng/mL)	97.08 ± 10.14	146.85 ± 25.42	70.86 ± 5.11
Insulin (mU/L)	11.79 ± 3.18	21.61 ± 6.19	17.9 ± 2.69
**Antiaging biomarker**			
Klotho (pg/mL)	2.25 ± 0.04	10.61 ± 0.56***	13.52 ± 0.64***

Data were analyzed using one-way ANOVA and post-hoc analysis performed by Tukey's test. At the end of study, subjects present in the placebo group (n = 42) and TRI 360^TM^ group (n = 42).

^***^p ≤ 0.001 vs. Placebo control group.

#### Assessment of neurotransmitters

Tukey's *post-hoc* analysis assessed, serum acetylcholine level was significantly increased by 18.41% (at day 90) and 45.03% (F_(2, 123)_ = 9.170, *p* ≤ 0.001; at day 180) in the TRI 360^TM^ group with respect to the placebo group (3217.36 ± 63.14 pg/mL). Serum norepinephrine level was significantly (F_(2, 123)_ = 61.186, *p* ≤ 0.001) increased by 123.13% (at day 90) and 45.15% (at day 180) in the TRI 360^TM^ group with respect to the placebo group (4.54 ± 0.14 ng/mL). Besides, serum concentration of dopamine was significantly (F_(2, 123)_ = 168.673, *p* ≤ 0.001) increased by 184.59% (at day 90) and 379.86% (at day 180) in the TRI 360^TM^ group as compared to the placebo group (Table 4).

**Table 4:** Measurement of neurotransmitters (acetylcholine, noradrenaline, and dopamine) in serum after treatment with the TRI 360^TM^ in human subjects, measured at days 90 and 180.

**Parameter**	**Placebo** **(Mean ±SEM)**	**TRI 360** ^TM^	
		**Day 90** **(Mean ±SEM)**	**Day 180** **(Mean ±SEM)**
Acetylcholine (pg/mL)	*3, 217.36*±63.14	*3, 809.30*±265.72	*4, 666.25*±314.68***
Norepinephrine (ng/mL)	4.54 ± 0.14	10.13 ± 0.50***	6.59 ± 0.35***
Dopamine (pg/mL)	382.44 ± 6.47	*1, 088.38*±91.10***	*1, 835.16*±32.33***

Data were analyzed using one-way ANOVA and post-hoc analysis performed by Tukey's test. At the end of study, subjects present in the placebo group (n = 42) and TRI 360^TM^ group (n = 42).

^***^p ≤ 0.001 vs. Placebo control group.

#### Inflammatory biomarkers

Pair *t*-test analysis indicated the expression of tumor necrosis factor-alpha (TNF-α) was significantly (CI = 1.942 to 13.878; *t* = 2.637, *p* = 0.010) reduced by 99.87% (at day 180), while non-significantly reduced by 44.44% (at day 90) in treatment group than placebo (7.92 ± 3.00 pg/mL). Moreover, serum concentration of interleukin-1β (IL-1β) level was significantly (CI = 0.472 to 5.128; *t* = 2.393, *p* = 0.019) reduced by 98.94% (at day 180), while non-significantly reduced by 25.44% (at day 90) in treatment group compared to the placebo group. Further, the expression of IL-8 was significantly (F_(2, 123)_ = 9.381, *p* ≤ 0.001) decreased by 43.91% (at day 90) and 64.58% (at day 180) in the TRI 360^TM^ group as compared to the placebo group (Table 5).

**Table 5:** Measurement of inflammatory cytokines (TNF-**α**, IL-1**β**, and IL-8) in serum after treatment with the proprietary dietary supplement (TRI 360^TM^) capsules in human subjects, measured at days 90 and 180.

**Parameter** **(pg/mL)**	**Placebo** **(Mean ±SEM)**	**TRI 360** ^TM^	
		**Day 90** **(Mean ±SEM)**	**Day 180** **(Mean ±SEM)**
TNF-α	7.92 ± 3.00	4.40 ± 2.58	0.01 ± 0.01**
IL-1β	2.83 ± 1.17	2.11 ± 1.03	0.03 ± 0.02*
IL-8	18.24 ± 3.19	10.23 ± 0.87***	6.46 ± 0.80***

Data were analyzed using one-way ANOVA and post-hoc analysis performed by Tukey's test. At the end of study, subjects present in the placebo group (n = 42) and TRI 360^TM^ group (n = 42);

^*^p ≤ 0.05,

^**^p ≤ 0.01 and ***p ≤ 0.001 vs. Placebo control group.

## Discussion

The large-scale clinical trial is a real-world effectiveness of the effect of dietary supplement, TRI 360^TM^ capsules on mental disorders such as physical tiredness, fatigue, psychological symptoms like depression, cognition, mental restlessness, etc., in human subjects. The authors also found an excellent correlation between changes in psychological symptoms and physiological biomarkers with respect to the placebo control subjects. This study is one of the proofs of concept study conducted to examine the outcomes of dietary nutrients like essential vital vitamins, minerals, and ginseng extract powder on mental health. This study reports on the first randomized clinical trial to explore a causative association between dietary supplementation with psychological and mood disorders. Treatment group participants in this trial received TRI 360^TM^ capsule twice daily for 180 days. In the present study, authors observed positive changes in the perceived psychological scorings on fatigue, sleep disturbances, depression, stress and confusion, mental restlessness, future fearness, emotional trauma, hopelessness, libido/sexual desireness, mood disorders symptoms, willpower, motivation, were more favorable in the TRI 360^TM^ group as compared with the placebo group. This study has shown the evidence of benefits of TRI 360^TM^ supplementation across a broad range of psychological and mental health symptoms. Moreover, numerous studies indicated the nutritional deficiencies of minerals (zinc, magnesium, iron, and selenium), vitamins, and amino acids are common in mental health disorders (33). TRI 360^TM^ may have a protective effect against the development of various psychological/mental health symptoms. This trial demonstrated that continuous intake of TRI 360^TM^ novel proprietary capsules led to improvements in vigor, tension, stress, anxiety, sleep quality, and physical movements. The possibility was also considered that this proprietary product could have self-medication effects on people with mood disorders and emotional problems. We also hypothesized that increased levels of vitamins- vitamin C and D_3_ metabolites (25 dihydroxy vitamin D_3_ and 1, 25 dihydroxy vitamin D_3_), hormones (oxytocin, 17-β-estradiol, and insulin), neurotransmitters (acetylcholine, noradrenaline, and dopamine), antiaging protein (klotho), and decreased levels of proinflammatory cytokines (TNF-α, IL-1β, and IL-8), and oxidative stress biomarker (oxidized-LDL, malondialdehyde) might be involved in the pathophysiology of psychiatric symptoms. We found a link between psychological scores and the various physiological biomarkers levels.

Vitamins like cholecalciferol (D_3_) plays a vital role in mental health and cognitive functions and regulates emotions and behavior. Recent studies confirm vitamin D deficiency's link to dementia, psychosis, autism, and cognitive decline (16). The literature has been reported that the inadequate intakes of specific nutrients such as folate, vitamin B_12_, selenium, iron, and zinc lead to mental disorders like depression, anxiety, etc., (17). In this trial, the proprietary dietary supplement (TRI 360^TM^) capsules significantly increased the level of more bioavailable vitamin C and vitamin D_3_ metabolites (25-(OH)_2_D_3_ and 1,25-(OH)_2_D_3_) in serum, which might be beneficial for the improvement of overall physical stamina/energy and immunity in immune deficient subjects, psychiatric population, and mental health disorders.

Numerous studies have reported that oxidation and glycation stresses are the main factors of aging-related chronic diseases and neurodegenerative diseases. These stresses produce a toxic intermediate, unsaturated carbonyl, such as malondialdehyde (MDA). Based on the study by Talarowska et al., an elevated levels of MDA were associated with delays in recall of declarative memories, short-term working memory, and visual-spatial working memory in patients with recurrent depression disorders (RDD) (26). After taking TRI 360^TM^, most of the subjects showed significantly decreased levels of MDA. Many mental disorders, such as depression, anxiety, schizophrenia, and bipolar disorder, are caused by oxidative stress (27). Our findings suggest that the levels of MDA and oxidized LDL in serum were significantly decreased in the nutraceutical combination subjects compared to placebo group, which could be helpful to the stress/depressed patients.

Oxytocin regulates human social behavior, including social decision-making, responding to social stimuli, forming social memories, and mediating social interactions. Moreover, it is also used in multiple psychiatric disorders such as autism, schizophrenia, and mood and anxiety disorders (18). Subjects in the nutraceutical combination group showed a significant elevation of love-making neuro-hormone, oxytocin in serum in adult subjects (female), compared with the placebo control group, which might be responsible for improving psychological symptoms like mood alleviation, calmness of mind, cognition, mental strength, etc.

Klotho is a pleiotropic hormone that controls the aging process and enhances body and brain health. Several studies reported that high chronic stress leads to lower levels of klotho. Overexpression of klotho enhances learning and memory ability and reverse synaptic and cognitive impairments in the animal experiment (24). Our findings suggest that the level of klotho protein in serum was significantly increased in the dietary and nutraceutical supplement subjects compared to placebo. The higher level of antiaging protein klotho might be helpful for the improvement of cognition, memory, and quality of life in adult subjects.

Numerous study findings showed that mental illnesses result from problems with the communication between neurons in the brain (*i.e.*, neurotransmission). Both animal and human studies showed that a decrease in acetylcholine (Ach) causes cognition deficits in Alzheimer's disease. Conversely, patients taking medications that reduce the breakdown of Ach and improve memory functions (21). Norepinephrine (NE) plays a vital role in behavioral and psychological symptoms, including depression, aggression, agitation, and psychosis (22). Dopamine plays a pivotal role in reward prediction, motivational arousal, and responsiveness to conditioned incentive stimuli (23). In this trial, the results showed that the nutraceutical combination treatment group participants significantly increased the level of the neurotransmitters *viz*. acetylcholine, norepinephrine, and dopamine in serum, which might be beneficial for depressed populations and mental health disorders and might be helpful for the improvement of cognition, memory, and quality of life in adult subjects.

Growing evidence suggests that proinflammatory cytokines are involved in the pathophysiology of many psychiatric conditions such as sleep disturbance, anxiety, depression, post-traumatic stress disorder, stress, and mental restlessness in adults associated with inflammation (25). One of the objectives of this study was to find out the correlation between psychiatric symptoms and proinflammatory cytokines in the serum sample of human subjects using a broad index of psychiatric symptoms. Dietary and nutraceutical combination treatment group showed a significantly lowered level of IL-8 in serum compared to placebo, which might support the treatment of chronic inflammatory disorders (arthritis, diabetes, obesity, and osteoporosis) and common mental disorders (CMDs) *i.e.*, depression, anxiety disorders, insomnia, and stress-related symptoms.

This study has both strengths and limitations. To our best knowledge, this is the first clinical study investigated the association between consumption of nutraceutical combination and different physiological biomarkers, which are substantial contributors to the pathogenesis of stress, anxiety, depression and many other mental disorders. Other strengths include successfully implementing of a robust experimental design devised to collect long-term and comprehensive data in a prospective clinical study. An additional strength was excellent adherence by the participants, as demonstrated by the data. Apart from novel positive outcomes, this trial have some limitations include low sample size and single-center. The mechanisms of the effects of nutraceutical combination observed in this study have not been fully revealed. Therefore, it will be necessary to conduct mechanistic studies on a larger population to examine the benefits of TRI 360^TM^ on mental health disorders. The well-controlled double-blind experiment is needed to further verify these results. Unfortunately, because of limited funding, we did not fulfill these limitations at this juncture.

## Conclusion

The proprietary nutraceutical combination product (TRI 360^TM^) was found to be as safe, tolerable, and free from any adverse reactions for long-term use in adult subjects. Overall, these findings support the daily intake of TRI 360^TM^ supplementation as a potentially safe dietary supplement for improving perceived psychological symptoms related to fatigue, stress, mental restlessness, memory problem, anxiety, depression, emotional and mood disturbances, sleep disorders, and improving the health-related quality of life in healthy populations, at the dosage used herein. This novel development may help in the design of specific nutraceutical combination formulation that are more beneficial in the regulation of psychological problems. It is clear that this nutraceutical combination may have different other therapeutic values which should be subjected to further rigorous experiments to clarify better clinical benefits. This is a very fruitful and exciting area for future research.

## Data availability statement

The original contributions presented in the study are included in the article/Supplementary material, further inquiries can be directed to the corresponding author.

## Ethics statement

The studies involving human participants were reviewed and approved by Sangini Hospital Ethics Committee, Gujarat, India (Reg. No. ECR/147/Inst/GJ/2013/RR-16). The patients/participants provided their written informed consent to participate in this study. Informed consent was obtained from all subjects involved in the study. Each subject provided written informed consent before intervention begun and after the subject has been informed of the benefits and risks of the protocol. The clinical study protocol, informed consent document and all other relevant study documentation were reviewed and approved by the responsible Ethics Committee (EC). The study protocol and consent forms have been approved by the medical and animal experiment ethic committee of Sangini Hospital Ethics Committee, Gujarat, India.

## Author contributions

MT contributed with regards to the concept and design of the study. SM wrote the manuscript, analysis, and interpreted the data. AB reviewed the draft manuscript. DT reviewed the manuscript, data compilation, and crosscheck. SJ contributed to study design, revised the manuscript critically for important intellectual content, and gave final approval of the version to be published. All authors reviewed the manuscript.

## Conflict of interest

Authors MT, AB, and DT were employed by Trivedi Global, Inc. Authors SM and SJ were employed by Trivedi Science Research Laboratory Pvt. Ltd.

## Publisher's note

All claims expressed in this article are solely those of the authors and do not necessarily represent those of their affiliated organizations, or those of the publisher, the editors and the reviewers. Any product that may be evaluated in this article, or claim that may be made by its manufacturer, is not guaranteed or endorsed by the publisher.

## Footnotes

^1^https://www.regulations.gov; https://www.ecfr.gov/cgi-bin/textidx?SID=e956d645a8b4e6b3e34e4e5d1b690209andmc=trueandnode=pt21.3.184andrgn=div5.

^2^ https://www.meddra.org

## Abbreviations

RCT: Randomized controlled trial
MDA: Malondialdehyde
LDL: Low density lipoprotein
BMI: Body mass index
AGE: Advanced glycation end products
RDA: Recommended daily allowance
GRAS: generally regarded as safe
eCRF: Electronic case report forms
AEs: Adverse effects
MedDRA: Medical Dictionary for Regulatory Activities
PQS: Psychological Questionnaire Scoring
PTSD: Post-traumatic stress disorder
ADD: Attention deficit disorders
ADHD: Attention deficit hyperactivity disorders.
