# Supplementary material for: Efficacy of a novel proprietary dietary supplement (TRI 360TM) on psychological symptoms and stress-related quality of life in adult subjects: A randomized controlled clinical trial
Source: Front Psychiatry. 2022 Aug 11;13:919284. doi: 10.3389/fpsyt.2022.919284 (PMC9403741; doi:10.3389/fpsyt.2022.919284)
Supplement: Supplementary file 1 [file Table_1.DOCX]

**Annexure 1**

| **Areas / Questions** | **Rarely**  **(1)** | **Some- times**  **(2)** | **Periodic**  **(3)** | **Frequently**  **(4)** | **More Frequent**  **(5)** | **Most Frequent (6)** | **Continuously (7)** |
| --- | --- | --- | --- | --- | --- | --- | --- |
| **Asthenia (general weakness/tiredness/fatigue)** | | | | | | | |
| Do you feel exhausted all the time? |  |  |  |  |  |  |  |
| Do you suffer from low energy or tiredness? |  |  |  |  |  |  |  |
| **Sleep disturbances (poor quality sleep)** | | | | | | | |
| How is your sleep? |  |  |  |  |  |  |  |
| How is the quality of your skin? |  |  |  |  |  |  |  |
| **Anxiety/Depression /PTSD** | | | | | | | |
| Are you anxious all the time or sometimes? |  |  |  |  |  |  |  |
| Are any incidences in your past always disturbing you? |  |  |  |  |  |  |  |
| **Stress and Confusion** | | | | | | | |
| Are you under stress in terms of your health, finances, education of your children, job or business of your husband/yourself, relationship, etc. |  |  |  |  |  |  |  |
| Do you feel bored, lost or confused in life? |  |  |  |  |  |  |  |
| **Mental Restlessness/mind chattering/distress** | | | | | | | |
| Are you always busy with lots of unwanted thoughts? |  |  |  |  |  |  |  |
| Do you always talk to yourself? |  |  |  |  |  |  |  |
| **Future Fear/ongoing negative thoughts** | | | | | | | |
| Do you and/or your spouse experience dissatisfaction with your job or business? |  |  |  |  |  |  |  |
| Do you experience dissatisfaction with your family's monthly income? |  |  |  |  |  |  |  |
| **Emotional Trauma** | | | | | | | |
| Do you feel unhappiness or sadness in your life? |  |  |  |  |  |  |  |
| Do you suffer from dissatisfaction or frustration in your life? |  |  |  |  |  |  |  |
| **Lack of self-worth (self-esteem)** | | | | | | | |
| Do you perceive yourself as a successful person? |  |  |  |  |  |  |  |
| Do you believe nobody understands you? |  |  |  |  |  |  |  |
| **Hopelessness / Suicidal Ideation** | | | | | | | |
| Do you feel your life is filled with sadness? |  |  |  |  |  |  |  |
| Do you lack purpose or meaning in life? |  |  |  |  |  |  |  |
| **ADD/ADHD (Inability to focus)** | | | | | | | |
| Can you focus on your work? |  |  |  |  |  |  |  |
| Are you easily distracted? |  |  |  |  |  |  |  |
| **Libido/sexual desireness** | | | | | | | |
| Do you feel disinterested in any kind of sexual activities? |  |  |  |  |  |  |  |
| Do you feel your libido is low? |  |  |  |  |  |  |  |
| **Menstrual/Mood disorders symptoms (female subjects)** | | | | | | | |
| Do you have irregular periods, heavy bleeding, low bleeding or spotting? |  |  |  |  |  |  |  |
| Do you experience bloating, cramps, headaches, or aches or pain in back, abdomen or muscles during menstruation? |  |  |  |  |  |  |  |
| **Low confidence/low willpower/inability to make the right decision** | | | | | | | |
| Do you experience inability to focus? |  |  |  |  |  |  |  |
| Do you feel low confidence? |  |  |  |  |  |  |  |
| **Lack of inspiration/Motivation/Enthusiasm** | | | | | | | |
| Do you experience lack of inspiration, motivation, enthusiasm or ambition in your life? |  |  |  |  |  |  |  |
| Do you have desire to become independent and do something big for your family? |  |  |  |  |  |  |  |
